# Repair bond strength of composite: Effect of surface treatment and type of composite

**DOI:** 10.4317/jced.54030

**Published:** 2018-06-01

**Authors:** Maryam Ghavam, Maryam Naeemi, Sedighe-Sadat Hashemikamangar, Hooman Ebrahimi, Mohammad-Javad Kharazifard

**Affiliations:** 1 DDS, MS, Associate professor, Department of operative dentistry, Dental school, Tehran University of Medical Sciences, International campus, Navab St. Tehran, Iran; 2 Dental student, Dentist, Tehran University of Medical Sciences, International campus, Dental school, Navab St. Tehran, Iran; 3 DDS, MS, Associate Professor, Department of Pediatric dentistry, Dental school, Tehran University of Medical Sciences, International campus, Navab St. Tehran, Iran; 4 DDS, MS, Assistant professor, Tehran University of Medical Sciences, International campus, Navab St. Tehran, Iran; 5 DDS, Department of epidemiology and biostatistics, Faculty of public health, Tehran University of Medical Sciences, Kargar St. Tehran

## Abstract

**Background:**

By an increase in use of composite restorations, some defects are also seen in these restorations, which need to be repaired. Since complete replacement of an old restoration may compromise the tooth structure, repair of defect is a more practical approach if there is no caries recurrence. Risk of pulp injury also decreases as such. One major challenge in restoration repair is to obtain a durable bond between the new and old composite. Laser irradiation has been suggested for surface preparation of old composite. This study aimed to assess the effect of composite surface preparation with Er,Cr:YSGG laser on microtensile bond strength to new composite.

**Material and Methods:**

A total of 18 blocks were fabricated in three groups of nanohybrid, microhybrid and Beautiful II giomer measuring 4x7x7 mm and subjected to 10,000 thermal cycles between 5-55°C with 30 seconds of dwell time. The samples were randomly assigned to no surface treatment (etching and bonding) or laser plus etching and bonding groups. Composite cylinders measuring 4x7x7 mm were fabricated of Beautiful, nanohybrid and microhybrid composites on old composite surfaces and subjected to 500 thermal cycles for 50 seconds between 5-55°C with 30 seconds of dwell time. Each block was sectioned into 10 samples and they were subjected to microtensile bond strength test. Data were analyzed using ANOVA and Tukey’s test.

**Results:**

In all composites, the mean bond strength in laser subgroups was higher than that in control subgroups except for giomer, which showed lower bond strength in laser subgroup. The lowest mean bond strength was noted in repair of Z350XT with Z350XT when the surface of old composite was etched (10.92 MPa). The highest mean bond strength was noted in repair of Z250 with giomer when the old composite surface was irradiated with laser (30.55 MPa).

**Conclusions:**

Er,Cr:YSGG laser plus etching increased the bond strength in all groups except for giomer group, which showed a reduction in bond strength.

** Key words:**Composite resins, surface treatment, tensile bond strength, laser, er,cr:ysgg, giomer.

## Introduction

At present, composite restorations are highly popular in restorative dentistry due to optimal esthetics, the ability to bond to tooth structure and requiring less removal of dental structure. However, composite resins, similar to other restorative materials, undergo fracture and need to be repaired (1-3). Complete replacement of a failed or fractured composite restoration results in removal of etched enamel and further removal of tooth structure to enhance the bond to enamel (4). Moreover, complete removal of old restorations results in creation of a larger cavity, removal of sound dental substrate, higher risk of pulp injury, waste of time and higher cost (5,6). Thus, in some cases, a new composite is added to repair the old restoration as a common treatment choice (7). However, one major challenge in repair of old composite restorations is to obtain a strong bond between the new and old composite (8). Prognosis of old to new composite bond depends on several factors such as surface properties of the old composite and type of surface treatment (4,5,9,10). Surface roughening and mechanical porosities are required to enhance the bond of old to new composite because chemical bond due to double bonds would degrade after long-term clinical service of composite restorations in the oral cavity (1-7).

Several methods are available for old composite surface preparation to enhance the bond to new composite such as surface roughening by bur, acid etching by fluoric acid, air abrasion with aluminum oxide particles, use of silane and resin-based adhesive systems and different primers (10,11).

Despite several studies, no consensus has been reached on a repair method applicable to a wide range of composite resins. Thus, there is a need for assessment of the efficacy of different protocols for repair of composites (12).

In the recent years, many advances have been made in use of laser in dentistry. Considering the mechanism of action of laser, the use of erbium lasers is one suggested method for surface preparation. Erbium lasers are used for cavity preparation and caries removal (13). It has been reported that lased surfaces are rough with surface porosities that enhance the retention of resin restorative materials (14).

Since in the clinical setting, no information is often available on the type of old composite, this study aimed to assess the microtensile bond strength of a new composite to several types of aged composites after laser preparation to find the best repair strategy for composite restorations.

## Material and Methods

**Figure 1:**
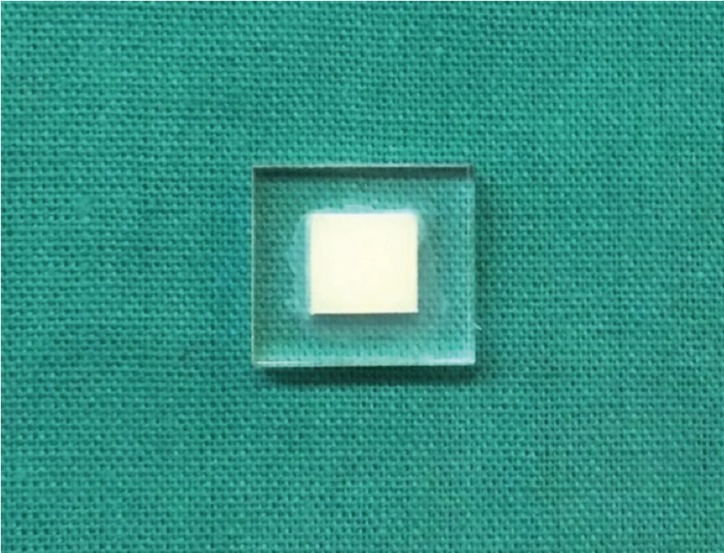
Fabricated composite sample measuring 7x7x4 mm.

In this *in vitro* experimental study, 18 blocks were fabricated of A2 shade dentin composites in three groups (n=6) of microhybrid (Z250), nanohybrid (Z350XT) and giomer (Beautiful II) ( Table 1). A prefabricated rectangular plexiform mold measuring 7x7 mm with 4 mm height was used for this purpose (Fig. 1). Petroleum jelly was applied to the mold and composite increments with 2 mm thickness were placed in the mold with a dental spatula and light cured for 20 seconds using a light curing unit (LIANGYA, LED, B200, Japan). At the time of curing, the tip of the light curing unit had 2 mm distance from the composite surface and was held perpendicular to it. The output of hand piece was measured by a radiometer to be 1000 mW/cm2. After the application of second layer of composite, a transparent Mylar strip was placed on the composite surface and a glass slab was placed over it with 1.5 mm thickness. The tip of the light curing unit was in contact with the slab and curing was performed for 20 seconds. The bottom surface of composite samples was also light cured for 20 seconds.

**Table 1:**
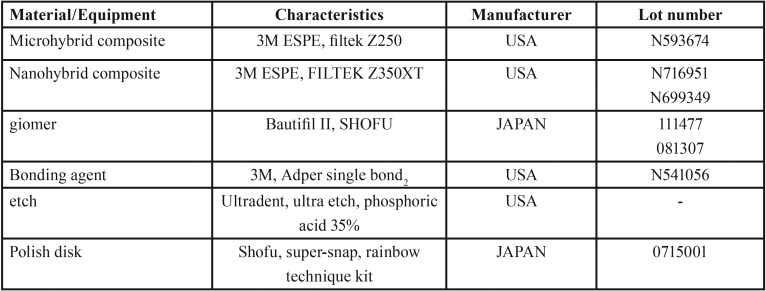
Characteristics of materials and equipment used in this study.

**Figure 2:**
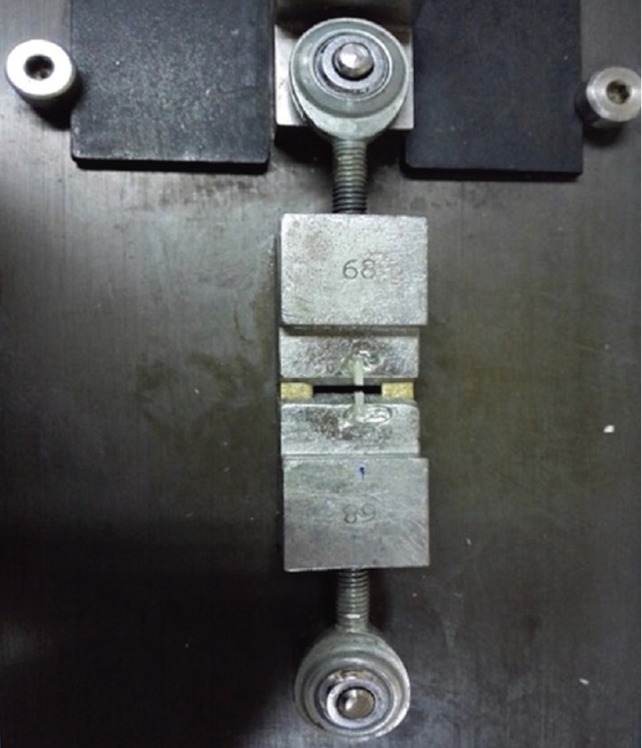
Microtensile tester.

The upper surface was polished with abrasive discs from coarse to fine (Shofu) for 30 seconds. New discs were used for each sample. The samples were then subjected to 10,000 thermal cycles between 5-55°C with 30 seconds of dwell time for aging. Next, of each group (n=6), three samples were randomly selected and subjected to Er,Cr:YSGG laser irradiation with 2780 nm wavelength, 20 mHz frequency, 3W power, 15 mJ energy and 119.42 J/cm2 energy density with 50% water and 60% air in contact mode. The distance from the surface was minimal and hand piece moved with a sweeping motion. The quartz mz tip with 800 µm diameter (Biolase-Waterlase) was used. All samples (18 blocks) were then etched with 35% phosphoric acid. Each block was etched for 15 seconds according to the manufacturer’s instructions and rinsed for 10 seconds and air dried. Adper Single Bond 2 was then applied by an applicator and rubbed on the surface for 15 seconds according to the manufacturer’s instructions. Air was sprayed for 5 seconds. The second layer of bonding agent was then applied and cured for 10 seconds. Next, Z250, Z350XT and giomer were randomly applied on samples in the subgroups and built up with the same dimensions as the underlying samples (7x7x4 mm). The fabricated 18 blocks were immersed in distilled water at 38°C for 24 hours and were then subjected to 500 thermal cycles between 5-55°C with 30 seconds of dwell time (TC300; Vafaie Industrial, Tehran, Iran). All samples were then mounted and sectioned by a mecatome (T201A). Ten samples were obtained of each block. A total of 180 samples were obtained and subjected to microtensile bond strength test (Bisco, USA) (Fig. 2). Data were analyzed using three-way ANOVA. Since all the interactions were significant, one-way ANOVA was used for comparison of all groups and Tukey’s post hoc test was applied to assess homogeneity of variances (*P*<0.05).

## Results

The mean, minimum and maximum microtensile bond strength between the old and new composites in 180 samples in two groups of laser and no laser are summarized in  Table 2. The results showed that the microtensile bond strength of Z250 and Z350XT composites in laser group was higher than that in no laser group; while this was reverse for giomer group.

**Table 2:**
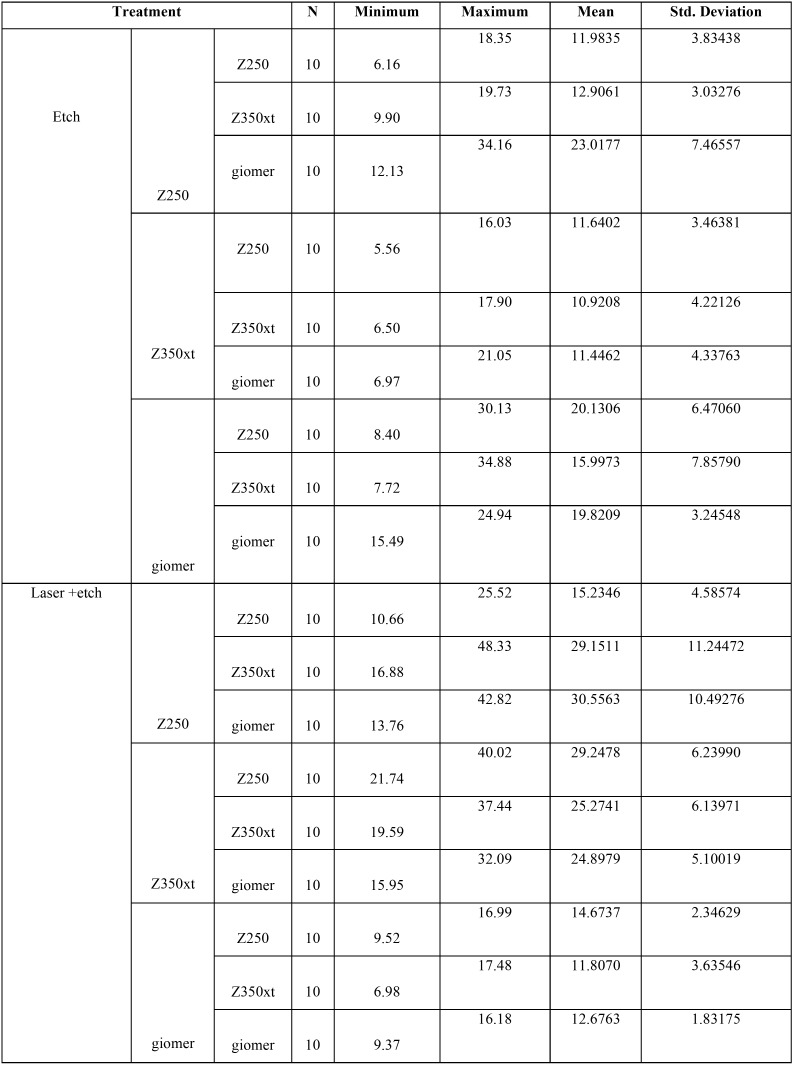
Mean, minimum and maximum microtensile bond strength between old and new composites in two groups of laser and no laser (n=10 for each subgroup).

The highest mean bond strength was noted in lased group of Z250 bonded to giomer (30.556 MPa) while the lowest mean bond strength was noted in non-lased group of Z350XT bonded to Z350XT (10.920 MPa).

One-way ANOVA showed a significant difference in use of Z250 as new composite in bond to different old composites in both lased (*P*=0.01) and non-lased (*P*=0.00) groups and an increase in bond strength was noted.

In use of giomer as new composite, significant differences were noted in bond to different old composites in both lased (*P*=0.00) and non-lased (*P*=0.00) groups and a reduction in bond strength was noted.

In bond of Z350XT as new composite, no significant difference was noted in bond strength to different old composites in non-lased group (*P*=0.129) but in lased group, the difference in bond strength among old composites was significant and a reduction in bond strength was noted (*P*=0.00,  Table 3).

**Table 3:**
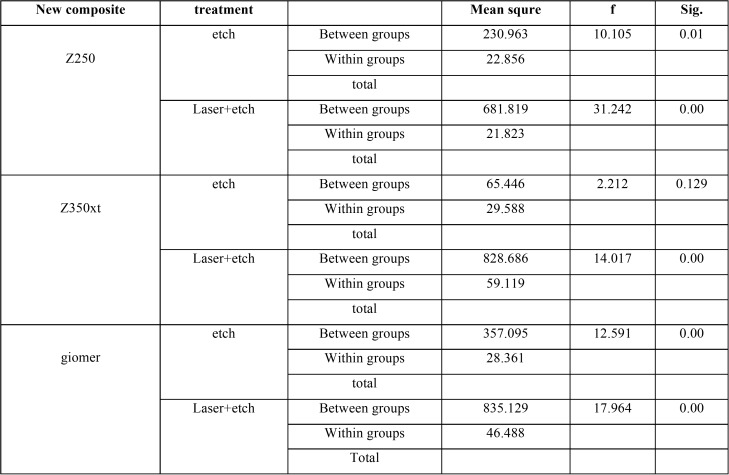
Results of one-way ANOVA.

Since the results of one-way ANOVA were significant, Tukey’s test was applied to assess the effect of type of new composite and surface treatment method on bond strength ( Table 4).

**Table 4:**
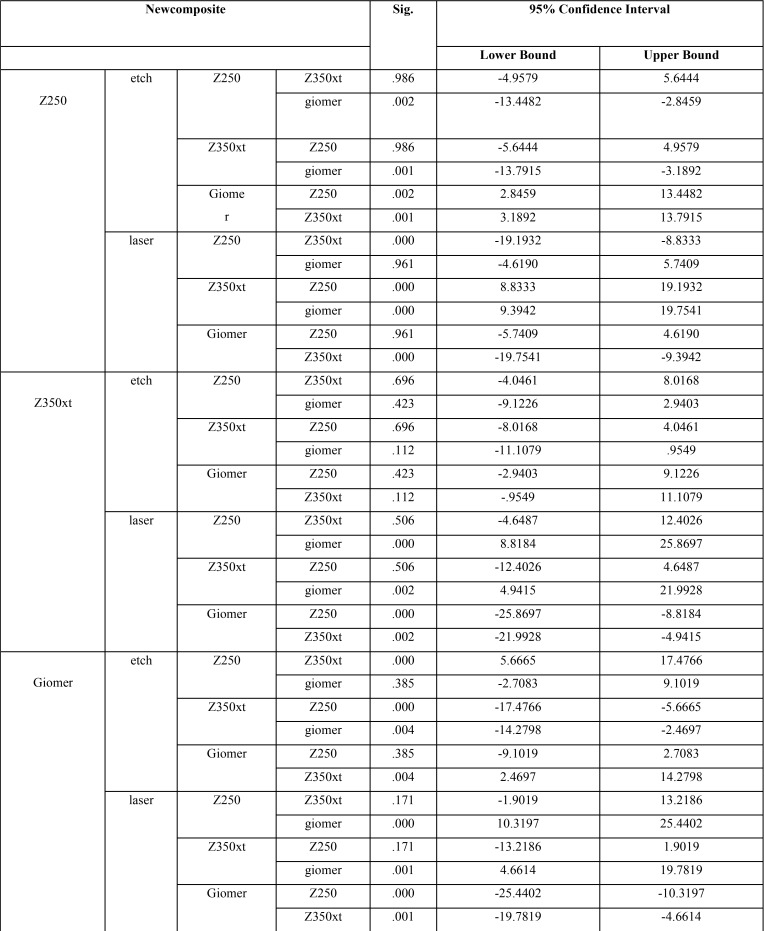
Tukey’s HSD test.

**Figure 3:**
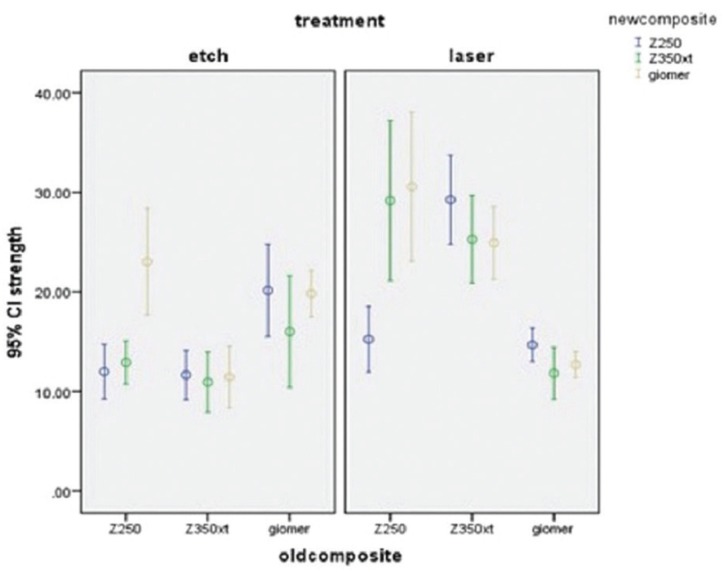
Microtensile bond strength between different composites in lased and non-lased groups.

Figure 3 shows the bond strength between different composites in lased and non-lased groups.

## Discussion

Replacement of restorations may yield superior clinical results and higher esthetics; however, it may also cause further destruction of tooth structure and pulpal injury while being time consuming and costly (2,10). Therefore, repair of composite restorations would be a more suitable option since it saves time, cost and the remaining tooth structure in comparison with complete replacement (1). However, repair of restorations may also be problematic. Roughening the surface may enhance a mechanical bond. Therefore, different mechanical and chemical surface treatments are performed to enhance the bond of old composite to new composite (1-7,10,11). In general, bond strength of old to new composite depends on factors such as aging conditions, time passed since the restoration of tooth, type of composite, surface roughness and type of bonding agent used (15).

No consensus has been reached on the most efficient method for aging. Several techniques have been used in previous studies for aging such as boiling, thermocycling and storage in citric acid, sodium chloride and distilled water. Water is the most commonly used medium for storage (11,16,17) but it has been reported that water storage can cause water sorption by the resin matrix and subsequent hydrolysis and release of filler particles. In the thermocycling process, water sorption can decrease the structural and physical properties of composite resins and cause aging of materials. In this study, 10,000 thermal cycles were performed between 5 to 55°C with a dwell time of 30 seconds. In this study, we did not aim to evaluate the effect of aging on composite resins; instead, we performed this process only for the purpose of standardization. Gale *et al.* (18) performed 10,000 thermal cycles *in vitro*. No consensus has been reached on the dwell time or number of cycles. Range of cycles has been reported from one to 1,000,000 with a mean value of 10,000 cycles. Nonetheless, Gale *et al.* performed 10,000 cycles corresponding to one year of clinical service in the oral environment (18).

Three commonly used types of composites were used in this study including a microhybrid (Z250), a nanohybrid (Z350XT) and a new type of composite known as giomer. The latter group of composite resins is made of Bis-GMA/TEGDMA. Giomers are direct restorative materials suitable for use in broken incisal edges, class V cavities, root surfaces, laminates and veneers; however, despite their increasing use as an esthetic restorative material, their bond strength has yet to be fully evaluated (19,20).

Previous studies evaluated the effect of micromechanical surface preparation by bur, sandblasting and acid etching on repair bond strength of composites (10,21). Another technique used for surface roughening is Er,Cr:YSGG laser irradiation (22,23). Our results showed that surface treatment with laser compared to etching in Z250 and Z350XT groups increased the microtensile bond strength; this result was in agreement with those of Kimiai *et al.*, (12) who reported positive effect of Er,Cr:YSGG laser on composite resins *in vitro* and stated that laser irradiation was the best method for repair of composite restorations *in vitro*. Mirzaei et al. (24) evaluated the effect of Er,Cr:YSGG laser on morphology of microhybrid composite under an electron microscope and reported that the increase in bond strength of composite in laser group compared to bur was due to creation of a micro-porous irregular surface. Also, Alizadeh *et al.* (25) used three types of lasers namely Er,Cr:YSGG, Nd:YAG and CO2 on silorane-based composites and discussed that Er,CR:YSGG laser was more effective than the other two (25).

Duran *et al.* (26) assessed the repair bond strength of composite using different levels of Er:YAG laser energy and discussed that laser can serve as an alternative to other surface treatments. However, as the laser energy increased, the composite bond strength decreased (26). In our study, laser irradiation decreased the microtensile bond strength of giomer, and the repair microtensile bond strength in etched group, irrespective of the type of composite, was higher than the bond strength in laser group. No previous study is available on the tensile bond strength of this composite except for a study by Arami *et al.* (27). They evaluated the effect of different surface treatment methods such as bur preparation, Nd:YAG laser and air abrasion on bond strength of giomer and showed that the bond provided by air abrasion was significantly higher than that of laser and the latter was higher than that of diamond bur preparation. However, in the study by kimyai *et al.*, (12) resin modified glass ionomers were subjected to surface treatment with laser and their results were not in accord with ours, which may be due to different types of composites used and their filler content.

The bond strength of old to new composites *in vitro* can be measured by shear or tensile tests. Fabrication of samples and standardization of their size for shear test is easier than that for microtensile test (28). However, assessment of shear bond strength is less reliable than assessment of tensile bond strength (29). Tensile bond strength test is often associated with fracture at the adhesive interface due to the uniform stress distribution. Fracture of material at the adhesive interface is more valuable than cohesive failure (30). Therefore, microtensile test was used in this study since we aimed to evaluate the bond strength of old to new composite at the interface. Acceptable repair bond strength of composites in the oral environment is still a matter of debate. However, acceptable bond of resin to enamel must be about 15 to 30 MPa (31,32); this value may also be acceptable in the clinical setting (31,33). Some authors believe that bond strength of composite must be higher than 18 MPa in order to be clinically acceptable. In this study, higher bond strength was noted in laser groups and no significant difference was noted between Z350XT nanohybrid and Z250 microhybrid composites, which was in line with the results of Nassoohi *et al.* (34). However, for giomer, the mean bond strength was lower than the acceptable threshold. Our results showed that not only the surface preparation method, but also the type of old and new composite played an important role in bond strength. After surface preparation with laser, bond strength of Z350XT nanohybrid to old Z250 microhybrid composite significantly increased; however, laser surface treatment was not effective for increasing the bond of Z250 to Z250 microhybrid composite. However, in the study by Nassoohi *et al.*, (34) a microhybrid and a nano-filled composite were compared and it was found that microhybrid composite yielded significantly higher bond strength than nano-filled composite (34). Our results showed that type of surface preparation had different effects on bond strength depending on the type of old and new composite.

However, some limitations exist against clinical use of laser for surface treatment. For instance, although Er,Cr:YSGG laser enhances the bond strength of nanohybrid and microhybrid composites, it requires special equipment and expertise of the operator. On the other hand, etching and bonding method is cost effective in the clinical setting and can be reliably used. This study had an *in vitro*, experimental design. Thus, future clinical studies are required to assess the effect of pH, thermal changes and saliva on bond strength.

## Conclusions

1. Er,Cr:YSGG laser was effective in all groups for increasing the bond strength but decreased the bond strength in giomer group.

2. Giomer can yield a high bond strength to Z250 old composite compared to other two composite resins.

3. All types of tested composites can be used for bond to Z350XT old composite with etching.

4. Laser irradiation significantly increased the bond of Z250 to Z350XT.

## Suggestions

Future *in vivo* studies are required using electron microscopy and different levels of laser energy as well as different types of laser. Also, since type of old composite can significantly affect the results, it is recommended to always write down the type of composite used for restorations in patients’ files for future reference.

## References

[1] Hasani Tabatabaei M, Alizade Y, Taalim S (2004). Effect of various surface treatment on repair strength of composite resin. Journal of Dentistry of Tehran University of Medical Sciences.

[2] Mjor IA (1993). Repair versus replacement of failed restorations. Int Dent J.

[3] Roberson TM, Heymann H, Sturdevant CM, Swift EJ (2006). Sturdevant's art and science of operative dentistry. 5th ed.

[4] Shahdad SA, Kennedy JG (1998). Bond strength of repaired anterior composite resins: an in vitro study. J Dent.

[5] Ozcan M, Barbosa SH, Melo RM, Galhano GA, Bottino MA (2007). Effect of surface conditioning methods on the microtensile bond strength of resin composite to composite after aging conditions. Dent Mater.

[6] Lucena-Martin C, Gonzalez-Lopez S, Navajas-Rodriguez de Mondelo JM (2001). The effect of various surface treatments and bonding agents on the repaired strength of heat-treated composites. J Prosthet Dent.

[7] Cavalcanti AN, Lavigne C, Fontes CM, Mathias P (2004). Microleakage at the composite-repair interface: effect of different adhesive systems. J Appl Oral Sci.

[8] Shahbazi M, Tabibi V (1997). Enamel and dentin Preparation for different dental material.

[9] Rinastiti M, Özcan M, Siswomihardjo W, Busscher HJ (2011). Effects of surface conditioning on repair bond strengths of non-aged and aged microhybrid, nanohybrid, and nanofilled composite resins. Clin Oral Investig.

[10] Bouschlicher MR, Reinhardt JW, Vargas MA (1997). Surface treatment techniques for resin composite repair. Am J Dent.

[11] Bonstein T, Garlapo D, Donarummo J Jr, Bush PJ (2005). Evaluation of varied repair protocols applied to aged composite resin. J Adhes Dent.

[12] Kimyai S, Mohammadi N, Navimipour EJ, Rikhtegaran S (2010). Comparison of the effect of three mechanical surface treatments on the repair bond strength of a laboratory composite. Photomed Laser Surg.

[13] Convissar RA (2011). Principle and practice of Laser Dentistry.

[14] de Souza AE, Corona SA, Dibb RG, Borsatto MC, Pecora JD (2004). Influence of Er:YAG laser on tensile bond strength of a self- etching system and a flowable resin in different dentin depths. J Dent.

[15] Fawzy AS, El-Askary FS, Amer MA (2008). Effect of surface treatments on the tensile bond strength of repaired water- aged anterior restorative micro-fine hybrid resin composite. J Dent.

[16] Ozel BO, Eren D, Herguner SS, Akin GE (2012). Effect of thermocycling on the bond strength of composite resin to bur and laser treated composite resin. Lasers Med Sci.

[17] Ozcan M, Cura C, Brendeke J (2010). Effect of aging conditions on the repair bond strength of a microhybrid and a nanohybrid resin composite. J Adhes Dent.

[18] Gale MS, Darvell BW (1999). Thermal cycling procedures for lab-oratory testing of dental restorations. J Dent.

[19] Tezvergil A, Lassila LV, Vallittu PK (2003). Composite-composite repair bond strength: effect of different adhesion primers. J Dent.

[20] Kukrer D, Gemalmaz D, Kuybulu EO, Bozkurt FO (2004). A pro- spective clinical study of ceromer inlays: results up to 53 months. Int J Prosthodont.

[21] Hannig C, Laubach S, Hahn P, Attin T (2006). Shear bond strength of repaired adhesive filling materials using different repair procedures. J Adhes Dent.

[22] Ozcan M, Barbosa SH, Melo RM, Galhano GA, Bottino MA (2007). Effect of surface conditioning methods on the micro- tensile bond strength of resin composite to composite after aging conditions. Dent Mater.

[23] Papacchini F, Dall'Oca S, Chieffi N, Goracci C, Sadek FT, Suh BI (2007). Composite-to-composite microtensile bond strength in the repair of a microfilled hybrid resin: effect of surface treatment and oxygen inhibition. J Adhes Dent.

[24] Mirzaei M, Yasini E, Tavakoli A, Chiniforush N (2015). Effect of Different Power of Er,Cr:YSGG Laser Treatment on Surface Morphology of Microhybride Composite Resin: Scanning Electron Microscope (SEM) Evaluation. J Lasers Med Sci.

[25] Alizadeh Oskoee P,  Mohammadi N,  Ebrahimi Chaharom ME,  Kimyai S,  Pournaghi Azar F,  Rikhtegaran S (2013). Effect of Surface Treatment with Er;Cr:YSSG, Nd:YAG, and CO2 Lasers on Repair Shear Bond Strength of a Silorane-based Composite Resin. Journal of Dental Research, Dental Clinics, Dental Prospects.

[26] İbrahim D, Çağrı U, Betül Y, Numan T (2015). Photomedicine and Laser Surgery. Mary Ann Liebert, Inc. publishers.

[27] Arami S, Kimyai S, Oskoee PA, Daneshpooy M, Rikhtegaran S, Bahari M (2017). Reparability of giomer using different mechanical sur- face treatments. J Clin Exp Dent.

[28] Rasmussen S (1996). Analysis of dental shear bond strength tests, shear or tensile. Int J Adhesion and Adhesives.

[29] Della BA, Anusavice K, Shen C (2000). Microtensile strength of composite bonded to hot-pressed ceramics. J Adhes Dent.

[30] Palasuk J, Platt JA, Cho SD, Levon JA, Brown DT, Hovijitra ST (2013). Effect of Surface Treatments on Microtensile Bond Strength of Repaired Aged Silorane Resin Composite. Oper Dent.

[31] Jafarzadeh Kashi TS, Erfan M, Rakhshan V, Aghabaigi N, Tabatabaei FS (2011). An in vitro assessment of the effects of three surface treatments on repair bond strength of aged composites. Oper Dent.

[32] Turner CW, Meiers JC (1993). Repair of an aged, contaminated indirect composite resin with a direct, visible-light-cured composite resin. Oper Dent.

[33] Teixeira EC, Bayne SC, Thompson JY, Ritter AV, Swift EJ (2005). Shear bond strength of self-etching bonding systems in combination with various composites used for repairing aged composites. J Adhes Dent.

[34] Nassoohi N, Kazemi H, Sadaghiani M, Mansouri M, Rakhshan V (2015). Effects of three surface conditioning techniques on repair bond strength of nanohybrid and nanofilled composites. Dent Res J.

